# Hepatic Stellate Cells Express Thymosin Beta 4 in Chronically Damaged Liver

**DOI:** 10.1371/journal.pone.0122758

**Published:** 2015-03-31

**Authors:** Jieun Kim, Sihyung Wang, Jeongeun Hyun, Steve S. Choi, Heejae Cha, Meesun Ock, Youngmi Jung

**Affiliations:** 1 Department of Integrated Biological Sciences, Pusan National University, Pusan, Korea; 2 Department of Biological Sciences, Pusan National University, Pusan, Korea; 3 Division of Gastroenterology, Department of Medicine, Duke University Medical Center, Durham, North Carolina, United States of America; 4 Department of Parasitology and Genetics, Kosin University College of Medicine, Pusan, Korea; Universite de Rennes 1, FRANCE

## Abstract

Although the various biological roles of thymosin β4 (Tβ4) have been studied widely, the effect of Tβ4 and Tβ4-expressing cells in the liver remains unclear. Therefore, we investigated the expression and function of Tβ4 in chronically damaged livers. CCl4 was injected into male mice to induce a model of chronic liver disease. Mice were sacrificed at 6 and 10 weeks after CCl4 treatment, and the livers were collected for biochemical analysis. The activated LX-2, human hepatic stellate cell (HSC) line, were transfected with Tβ4-specific siRNA and activation markers of HSCs were examined. Compared to HepG2, higher expression of Tβ4 in RNA and protein levels was detected in the activated LX-2. In addition, Tβ4 was up-regulated in human liver with advanced liver fibrosis. The expression of Tβ4 increased during mouse HSC activation. Tβ4 was also up-regulated and Tβ4-positive cells were co-localized with α-smooth muscle actin (α-SMA) in the livers of CCl4-treated mice, whereas such cells were rarely detected in the livers of corn-oil treated mice. The suppression of Tβ4 in LX-2 cells by siRNA induced the down-regulation of HSC activation-related genes, tgf-β, α-sma, collagen, and vimentin, and up-regulation of HSC inactivation markers, ppar-γ and gfap. Immunofluorescent staining detected rare co-expressing cells with Tβ4 and α-SMA in Tβ4 siRNA-transfected cells. In addition, cytoplasmic lipid droplets were observed in Tβ4 siRNA-treated cells. These results demonstrate that activated HSCs expressed Tβ4 in chronically damaged livers, and this endogenous expression of Tβ4 influenced HSC activation, indicating that Tβ4 might contribute to liver fibrosis by regulating HSC activation.

## Introduction

Liver fibrosis is the main characteristic of most chronic liver diseases. Hepatic stellate cells (HSCs) are known to be the major source of fibrous matrix production [1]. These cells undergo transdifferentiation from “quiescent” HSCs into “activated” HSCs during liver injury. The activated HSCs show a myofibroblast-like phenotype lacking cytoplasmic lipid droplets and having long processes. These fibrogenic myofibroblasts accumulate and promote the deposition of extracellular matrix proteins, leading to liver fibrosis [2]. Therefore, the study of the underlying mechanism of HSC activation has been considered to provide the important clues to develop the therapeutics for inhibiting liver fibrosis.

Thymosin β4 (Tβ4), a 43-residue acidic peptide, is the most abundant member of the highly conserved β-thymosin family. Tβ4 was first isolated from calf thymus and later identified as the major G-actin-sequestering protein in cells. It controls cell morphogenesis and motility by regulating the dynamics of the actin cytoskeleton [3]. Emerging evidence suggests that Tβ4 plays an important role in cancer progression, such as promoting angiogenesis, metastasis, and epithelial-to-mesenchymal transition [4,5]. The increased endogenous expression of Tβ4 has been reported in breast, ovarian, and uterine cancers, and Tβ4 has contributed to the metastasis in human colorectal, renal, and lung cancers [4,6–10]. In addition, Tβ4 was shown to prevent inflammation and fibrosis, promoting healing in the eye, skin, and heart [11–13]. The expression and function of Tβ4 have been investigated recently in the liver. Exogenous Tβ4 treatment has inhibited HSC activation and ameliorated the liver damage caused by a single injection of carbon tetrachloride (CCl_4_) [14–16]. However, it still remains unclear what type of cell expresses Tβ4. Nemolato et al. [17] reported that hepatocytes expressed Tβ4, whereas Paulussen et al. [18] provided the indirect evidence that CD 11b- or CD 68- positive cells might express Tβ4. Also, there has been no direct evidence that HSCs express endogenous Tβ4. Therefore, it was necessary to investigate the endogenous expression and function of Tβ4 in the liver in order to understand exactly the anti-fibrotic effect of exogenous Tβ4 in the damaged liver.

Herein, we assessed the expression of Tβ4 in the healthy and chronically damaged liver and showed the greater increase of Tβ4 in the damaged liver. It was first demonstrated that the activated HSCs expressed Tβ4, which regulated HSC activation.

## Materials and Methods

### Human samples, experimental cells and animals

The human hepatic stellate cell line LX-2, a well-characterized cell line derived from human HSCs [19], and HepG2 derived from the human hepatocellular carcinoma (HCC) were cultured in DMEM (HyClone, Logan, Utah, USA), supplemented with 10% fetal bovine serum and 1% penicillin-streptomycin at 37C in 5% CO_2_. HepG2 cell line is a well-recognized model system frequently used to investigate liver cell function in vitro [20]. LX-2 and HepG2 were friendly obtained from Dr. Jeong (Korea Advanced Institute of Science and Technology, Daejeon, Korea) and Dr. Kim (Pusan National University, Pusan, Korea) respectively. Human primary HSCs were purchased commercially from Zen-Bio Inc. (Durham, NC) and cells were grown in hepatic stellate growth medium (Zenbio Inc.) according to the manufacturer’s instructions. And human healthy liver tissues were generous gifts from Anna Mae Diehl and Steve S. Choi (Duke University Medical center), and used as normal controls. Those controls were obtained from residual healthy liver tissues of five donor livers that were utilized for split liver transplantation at Duke University Hospital. And these human liver samples used in this study have been described in previous publications [21,22]. Human fibrotic liver tissues with alcoholic hepatitis B were provided by National Biobank of Korea (PNUH, Pusan, Korea) (n = 13), and these samples were graded for the stage 4 of fibrosis. These chronic fibrotic tissues were surgically resected from livers with HCC and frozen at -70°C. The employed in these studies were all male. This study was approved by the local ethics committee (PNU IRB/2013_44_BR). Primary HSCs were isolated from normal C57BL/6 mice using standard approaches [23]. Freshly isolated HSC were used for RNA analysis or cultured on plastic dishes in serum-containing medium for 7 days. Male C57BL/6 mice at 6 weeks old were purchased from Hyochang (Dae-gu, Korea), fed with normal diet, watered, and housed with a 12h light-dark cycle. CCl_4_ chronic exposure is probably the most widely used model for studying chronic liver disease. The mouse model of liver fibrosis was induced by intraperitoneal injection of CCl_4_ (0.6ml/kg body weight) or corn oil vehicle twice per week as previously described [24]. Mice were sacrificed after 6 and 10 weeks of treatment. Liver tissue was either fixed in 10% neutral buffered formalin, frozen in O.C.T, or snap frozen in liquid nitrogen and stored at -80°C for histological and biochemical analysis. Animal care and surgical procedures were approved by the Pusan National University Institutional Animal Care and Use Committee and carried out in accordance with the provisions of the NIH Guide for the Care and Use of Laboratory Animals.

### RNA Isolation and quantitative RT-PCR

Total RNA was extracted with Trizol reagent (Life technologies, Inc.). After assuring RNA quality and concentration, total RNA (1μg) was used to synthesize cDNA using the SuperScript II First-strand Synthesis System (Invitrogen) following the manufacturer’s instructions. Gene expression was evaluated by QRT-PCR analysis. mRNAs were quantified by realtime RT-PCR using Power SYBR Green Master Mix (Applied Biosystem) according to the manufacturer’s specifications (Eppendorf Mastercycler, Real-Time PCR). Samples were analyzed in triplicate according to the Delta-Delta threshold (ΔΔCt) method. The following primer sequences were used for QPCR. Human Tβ4 primers, forward: CGC AGA CCA GAC TTC GCT CGT AC; reverse: TCC TTC CCT GCC AGC CAG ATA GAT; Human TGF-β primers, forward: GGG AAA TTG CTC GAC GAT; reverse: TTG ACT GAG TTG CGA TAA TGT T; Human α-sma primers, forward: AAT GGC TCT GGG CTC TGT AA; reverse: CTT TTC CAT GTC CCA GT; Human col1a1 primers, forward: TGT GAG GCC ACG CAT GAG; reverse: CAG ATC ACG TCA TCG CAC AA; Human vimentin primers, forward: CGA AAA CAC CCT GCA ATC TT; reverse: GTG AGG TCA GGC TTG GAA AC; Human PPAR-γ primers, forward: CGT GGC CGC AGA TTT GAA; and reverse: CTT CCA TTA CGG AGA GAT CCA C; Human GFAP primers, forward: CTG GAG GTT GAG AGG GAC AA; and reverse; CAG CCT CAG GTT GGT TTC AT; Human S9 primers, forward: GAC TCC GGA ACA AAC GTG AGG T; and reverse: CTT CAT CTT GCC CTC GTC CA; Mouse Tβ4 primers, forward: ATG TCT GAC AAA CCC GAT ATG GC; reverse: CAG CTT GCT TCT CTT GTT CA; Mouse S9 primers, forward: GAC TCC GGA ACA AAC GTG AGG T; and reverse: CTT CAT CTT GCC CTC GTC CA;. For analysis, according to the ΔΔCt method, each C_t_ value was first normalized to the respective 9S rRNA C_t_ value of the sample and afterward to the control. The fold induction was calculated from these C_t_ values. All PCR products were directly sequenced for genetic confirmation in Macrogen Inc (Korea).

### Western Blot

Total protein was extracted from frozen liver tissues and cultured cells. Samples were homogenized in Triton lysis buffer (TLB) supplemented with protease inhibitors (Roche). Equal amount of total protein were fractionated by SDS-PAGE on 15% tris-tricine gel as described by Hermann [25] and transferred to polyvinylidene difluoride (PVDF) membranes. Other detailed procedures were conducted as previously described [5]. Primary antibodies against Tβ4 (A9520, Immunodiagnostik AG, Bensheim, Germany) and GAPDH (MCA4739, AbDSerotec, Raleigh, NC) were used in this experiment. Membranes were developed by chemiluminescence (ATTO Corporation). The blots obtained from 3 independent experiments were scanned and an region of interest (ROI) around the band of interest was defined. Band intensities were calculated by using CS Analyzer Version 2.0 (ATTO Corporation).

### Liver histology and Immunohistochemistry

Liver specimens were fixed in 10% neutral buffered formalin, embedded in paraffin and cut into 4 μm sections. Specimens were deparaffinized, hydrated and stained usual method with standard hematoxylin and eosin (H&E) to examine morphology and Sirius Red to assess fibrosis. For immunohistochemistry (IHC), sections were incubated for 10 min in 3% hydrogen peroxide to block endogenous peroxidase. Heat-induced antigen retrieval was performed in 10 mM sodium citrate buffer (pH 6.0) for 10 min. Sections were blocked in Dako protein block (X9090; Dako, Carentaria, CA) for 30 min and incubated with primary antibody, Thymosin β4 (609270; Calbiochem, San Diego, CA) at 4°C overnight. Other sections were also incubated at 4°C overnight in non-immune sera to demonstrate staining specificity. Polymer horseradish peroxidase (HRP) anti-rabbit (K4003; Dako) was used as secondary antibody. 3,3′-Diaminobenzidine (DAB) was employed in the detection procedure. For double immunofluorescent staining, the frozen liver sections or cells were employed. Samples were fixed in and permeablized with acetone and methanol, respectively. They were washed with TBS and incubated with blocking solution for 30 minutes. Section or cells were incubated with primary antibody, Tβ4 (Calbiochem) and α-SMA (A5228; Sigma-Aldrich, St. Louis, MO, USA) for 4°C overnight. Other sections were also incubated at 4°C overnight in non-immune sera. The fluorescein labelled anti-rabbit IgG (Alexa Fluor 568, Invitrogen, Carlsbad, CA) and anti-mouse IgG (Alexa Fluor 488, Invitrogen) were used as secondary antibodies 30 min. 4’,6-diamidino-2-phenylinole (DAPI) were employed in the counterstaining procedure. In Oil Red O staining, cytoplasmic fat droplets were stained with 0.5% (wt/vol) Oil Red O (Sigma-Aldrich) in propylene glycol.

### Tβ4 siRNA transfection

siRNA was used for gene knockdown studies. LX-2 cells and human primary HSCs were cultured in six-well plates. After incubation overnight to allow cell attachment, cells were serum-starved for 24 hours. Then, cells were cultured in antibiotics-free medium for 24h prior to transfection. Cells were transfected with 25nM of Tβ4 siRNA (a mixture of four siRNAs, SMART-pools, Dharmacon, Lafayette CO, USA) or negative control siRNA (non-targeting siRNA, siCONTROL, Dharmacon) for 24h using Lipofectamine RNAiMAX reagents (Invitrogen) in serum-free Opti-MEMI (Invitrogen) according to the manufacturer’s instructions. After control or Tβ4 siRNA transfection, cell viability was determined to be > 90%, as established by trypan blue exclusion. Cells were harvested and total RNA was extracted for gene expression analysis. The efficiency of gene knock down was evaluated by QRT-PCR. Protein knockdown by siRNA was confirmed by Western Blot analysis with specific antibody.

### Cell counting

To qualify the number of Tβ4-positive cells, 10 areas of the histological slides were randomly selected per section at X40 magnification for each mouse liver. The Tβ4-positive cells were quantified by counting the total number of Tβ4-positive cells per field and dividing by the total number of hepatocytes per field. The percentage of Oil Red O (ORO)–positive cells was determined by counting ORO-positive cells per field in 20 randomly selected pictures from each group. Cells staining positively for Oil Red O (ORO) in 20 randomly selected fields per each group were counted at X40 magnification. The average number of ORO-positive cells was obtained by dividing the total number of positive cells by the total number of cells.

### Prolyl oligopeptidase (POP) activity

The POP activity assay was performed as described earlier [26]. Frozen sample (100 mg) was homogenized in 500 μl assay buffer (10mM Tris-HCl buffer, pH 7.4). The lysate was centrifuged at 16,000 g, for 20 min, at 4°C. The supernatant (10 μl) was incubated with 465 μl of assay buffer for 30 min, at 30°C. The reaction was initiated by adding 25 μl of substrate (4mM Suc-Gly-Pro-AMC, Bachem, Germany) was added. The reaction was allowed to proceed for 60 min at 30°C and terminated by the addition of 500 μl of 1 M sodium acetate buffer (pH 4.2). The formation of AMC was determined by measuring the fluorescence intensity at 460 nm with excitation at 360nm using GloMax^®^multi-detection system (Promega, Madison, WI). The results were expressed as picomole of AMC per minute per milligram of total protein.

### Statistical Analysis

Results are expressed as the mean±SD. Statistical differences were determined by Student’s t-test. P-values <0.05 were considered to be statistically significant.

## Results

### Expression of Tβ4 activated HSCs

**Fig 1:**
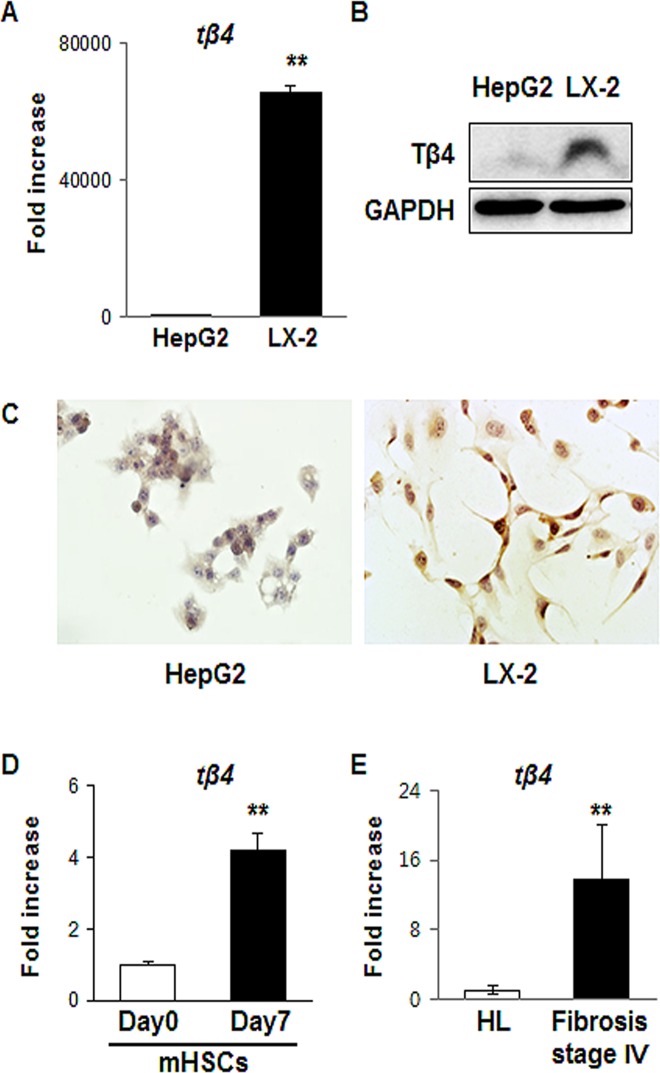
Detection of Tβ4 in the activated HSCs and human fibrotic liver. (A) QRT-PCR analysis of mRNA form human hepatic stellate cell line (LX-2) and human hepatocellular carcinoma cell line (HepG2). Mean±SD results are graphed. Data represent the mean±SD of three independent experiments (**p<0.005 vs. HepG2). (B) Western blot analysis of Tβ4 (GAPDH was used as an internal control). Data shown represent one of three experiments with similar results. (C) Representative immunostaining images for Tβ4 in LX-2 cells and HepG2 cells. Brown color indicated Tβ4-positive cells. (X40) (D) QRT-PCR analysis of mRNA from mouse primary HSCs (Day 0: freshly isolated HSCs / Day 7: cultured primary HSCs for 7 days. Mean±SD results are graphed. Data represent the mean±SD of three independent experiments (**p<0.005). (E) QRT-PCR analysis of RNA isolated from healthy livers (HL, n = 3) and fibrotic livers (stage 4 of fibrosis, n = 13). Mean±SD results are graphed and data represent the mean±SD of three independent experiments (**p<0.005).

To probe the association of endogenous Tβ4 with HSCs, we examined whether HSCs expressed Tβ4. The expression of Tβ4 in RNA level significantly increased in LX-2 cells, the activated human HSC line, compared with HepG2 cells (65860- ± 1458-fold increase, **p <0.005) (Fig 1A). Western blot analysis showed that LX-2 cells had a greater amount of Tβ4 protein than the HepG2 cells (Fig 1B). Also, immunostaining data demonstrated that LX-2 cells expressed Tβ4, which was rarely detected in HepG2 cells (Fig 1C). HSCs from the livers of healthy mice were isolated by using standard techniques and cultured for 7 days. Primary HSCs were known to be activated during culture [27–30]. RNA expression of Tβ4 was higher in cells cultured for 7 days than freshly isolated cells (Fig 1D). In addition, mRNA levels of Tβ4 were highly up-regulated in human livers with the stage 4 of fibrosis than healthy liver (13.723- ± 6.393-fold increase, **p <0.005) (Fig 1E). These data indicated that activated HSCs expressed Tβ4.

### Increased expression of Tβ4 in chronically damaged liver

**Fig 2:**
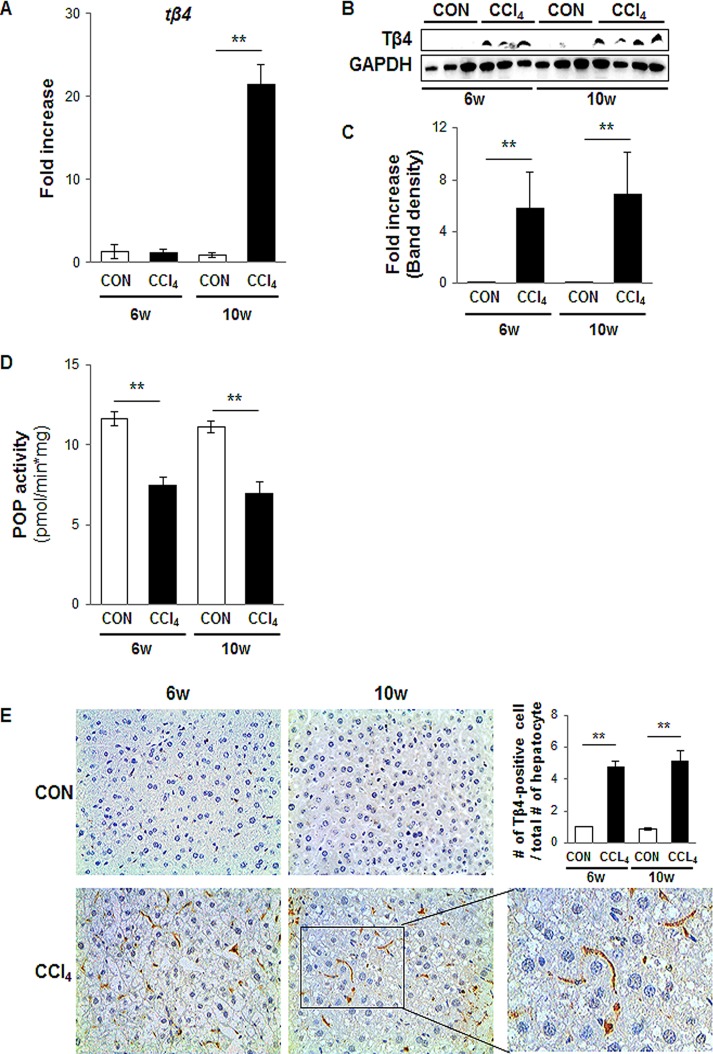
Up-regulation of Tβ4 in the livers from CCl_4_-treated mice. (A) QRT-PCR analysis of liver mRNA from CCl_4_-treated mice for Tβ4 (n = 4 mice / group). Mean±SD results are graphed. (B) and (C) Western blot analysis of Tβ4 (GAPDH was used as an internal control). Data shown represent one of three experiments with similar results. (B: Immunoblot / C: Band density) (n≥3 mice/ group) Data represent the mean±SD of three independent experiments (**p<0.005 vs. own control group). (D) POP activities in homogenates of controls and CCl4-treated mice are presented in picomole of AMC/min x mg tissue. Mean±SD results are graphed and data represent the mean±SD of three independent experiments (**p<0.005 vs. own control group). (E) IHC for Tβ4 in the liver at 6 and 10 weeks post CCl_4_ injection. Brown color indicated Tβ4-positive cells (X40). Magnified representative images from CCl_4_-treated livers at 6 and 10 weeks (X63) is shown in right panel. TB4-positive cells were quantified by dividing of the total number of positive cells by the total numbers of hepatocytes per field. Mean±SD results are graphed (**p<0.005 vs. own control group). (CON: corn oil-treated mice, CCl_4_: CCl_4_-treated mice).

When the liver is injured, HSCs are activated and produce the fibril matrix, promoting liver fibrosis [31]. Because up-regulation of Tβ4 was shown in the activated HSCs, not the normal liver, we examined the endogenous expression of Tβ4 in intact animals. To induce chronic liver damage, mice were injected with CCl_4_ for 6 and 10 weeks. H&E staining showed chronically damaged morphology, and Sirius red staining indicated collagen deposition in the liver of CCl_4_-treated mice (S1 Fig). Quantitative analysis for RNA expression demonstrated a higher expression of Tβ4 in the damaged liver at 10 weeks (21.390 ± 2.440-fold increase, **p<0.005) (Fig 2A). Protein expression of Tβ4 significantly increased in CCl_4_-treated mice at both 6 and 10 weeks, whereas it was not detected in the control mice injected with corn oil (5.8- ± 3.1-fold and 7.1- ± 3.2-fold increase at 6 and 10 weeks, respectively, compared with control, **p <0.005) (Fig 2B–2C).

RNA expression of Tβ4 in CCl_4_-induced liver injury was similar in the corn oil-injected liver, although protein expression of Tβ4 in the damaged liver showed a higher increase than the control liver at 6 weeks. According to Chen et al. [32], the prolyl oligopeptidase (POP), which converted Tβ4 into Ac-SDKP, decreased in the chronically injured liver. Also, Myöhänen et al. [26] reported that the highest activities of POP were found in the normal liver. Thus, it is possible that the decreased activity of POP in CCl_4_-treated mice at 6 weeks contributes to the up-regulation of Tβ4 protein, and then the accumulated Tβ4 protein turn on own signaling pathway, eventually promoting Tβ4 production in both RNA and protein at 10 weeks. To assess this possibility, we measured the POP activity, which was significantly reduced in CCl_4_-treated mice at both 6 and 10 weeks compared to corn-oil treated mice (0.64 ± 0.034- and 0.62 ± 0.059-fold decrease at 6 and 10 weeks, respectively, compared with own control, **p <0.005) (Fig 2D). These findings supported our explanation for the increased level of Tβ4 protein at 6 weeks after CCl_4_ treatment.

**Fig 3:**
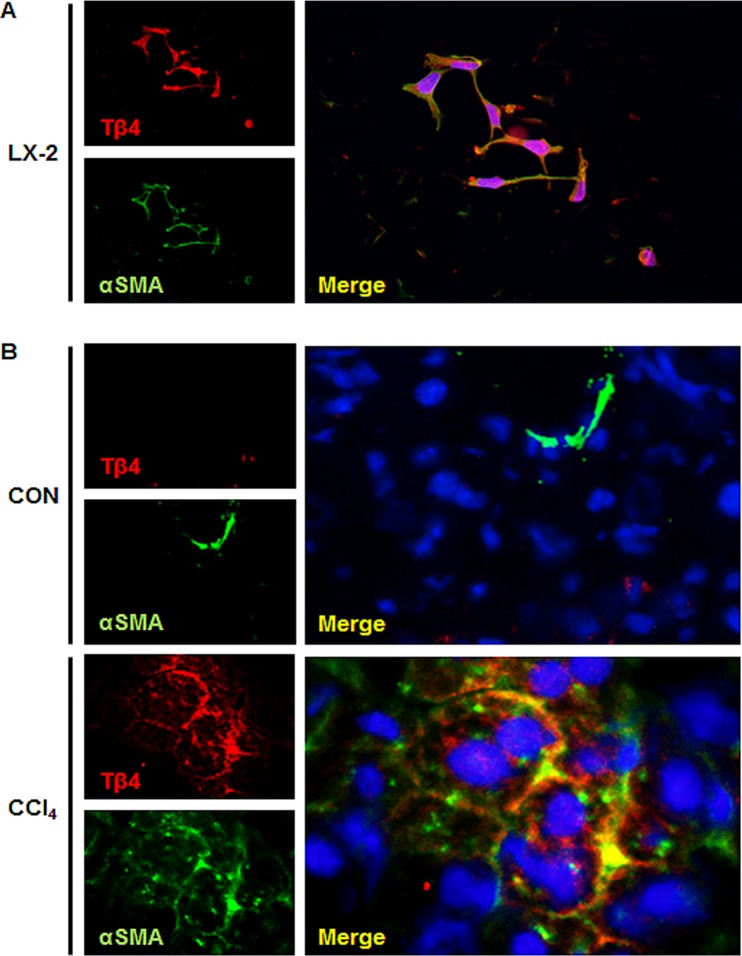
Co-localization of Tβ4 and α-SMA in the damaged livers. (A) Double immunofluorescent staining for Tβ4 and α-smooth muscle actin (α-SMA) in cultured LX-2 cells (X40). (B) Double immunofluorescent staining for Tβ4 and α-SMA in liver section from representative CCl_4_-treated mice and corn oil-treated mice (X63). Red and green colors indicate Tβ4 and α-SMA, respectively. Co-expressing cells are shown as yellow to orange. DAPI nuclear staining is shown as blue.

Immunostaining for Tβ4 confirmed the Western blot data by showing the accumulation of Tβ4-positive cells in the liver sections of mice that were treated with CCl_4_, whereas the corn oil-treated livers harbored only a few Tβ4-positive cells at 6 and 10 weeks (Fig 2E). The number of Tβ4-positive cells was significantly higher in CCl_4_-treated mice than corn oil-injected mice (4.74 ± 0.33- and 5.11 ± 0.67-fold increase at 6 and 10 weeks, respectively, compared with own control, **p <0.005). Tβ4 staining was more evident along fibrotic tracts, which were developed in the chronically damaged liver (S1 Fig). Because Tβ4-positive cells in the pericellular and perisinusoidal spaces had long processes extending into the spaces between adjacent hepatocytes, they looked like activated HSCs. To assess whether Tβ4-positive cells were the activated HSCs, we conducted double immunofluorescent staining for Tβ4 and α-smooth muscle actin (α-SMA), a fibrosis marker predominantly expressed in activated HSCs. The activated LX-2 cells co-expressed Tβ4 (red color) and α-SMA (green color). In addition, α-SMA-positive cells were co-localized with Tβ4-positive cells in the liver section from CCl_4_-treated mice (Fig 3). However, double positive cells for α-SMA and Tβ4 were rarely detected in corn oil-treated mice (Fig 3B). These data demonstrated that the expression of Tβ4 increased with the accumulation of activated HSCs, and those activated HSCs expressed Tβ4 in the chronically damaged mouse liver.

### Tβ4 regulates activation of HSCs

**Fig 4:**
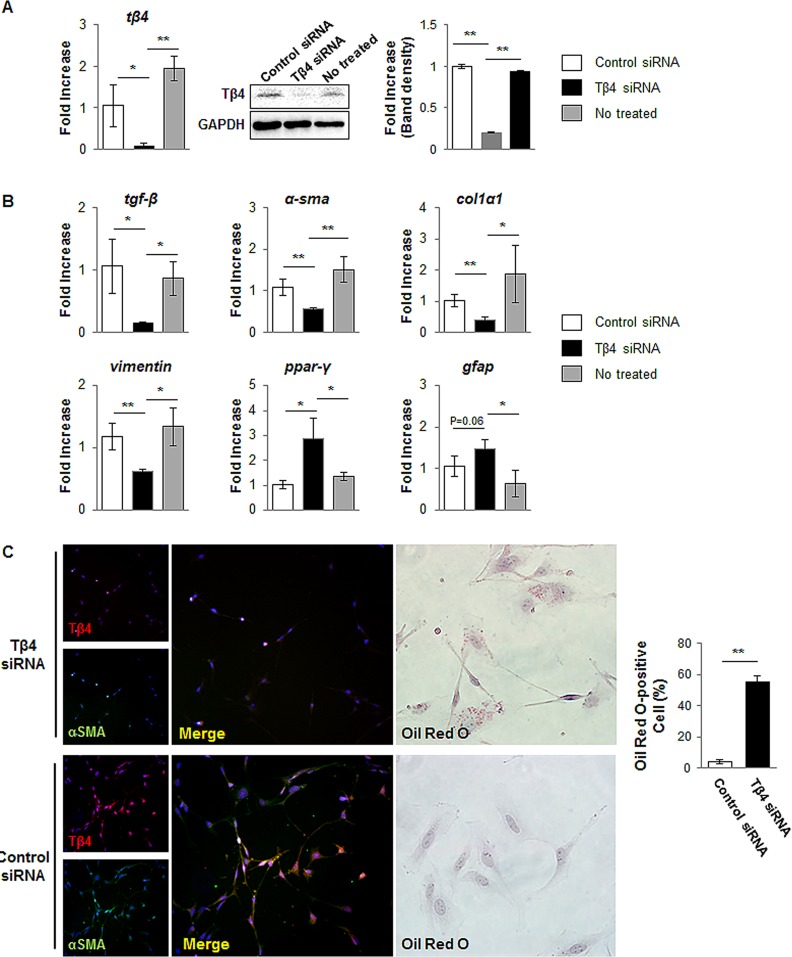
siRNA for Tβ4 blocks activation of LX-2 cells. (A) RNA and protein expression of Tβ4 in control siRNA-, Tβ4 siRNA- or non-treated LX-2 cells. Data represent the mean±SD of three independent experiments. GAPDH was used as an internal control (*p<0.05; **p<0.005). (B) QRT-PCR analysis for tgf-β, α-sma, col1α1, vimentin, ppar-γ and gfap. Mean±SD results are graphed. Data represent the mean±SD of three independent experiments (*p<0.05; **p<0.005 vs. control siRNA). (C) Double immunofluorescent staining for Tβ4 and α-SMA in Tβ4 siRNA-treated and control siRNA-treated cells (X20) and Oil Red O staining in siRNA-treated and control siRNA-treated cells (X40). ORO-positive cells were expressed as percentage of ORO-positive cells/total cells. Data represent the mean±SD of three independent experiments (**p<0.005). (Tβ4 siRNA: Tβ4 siRNA-treated LX-2 cells, Control siRNA: Control siRNA-treated LX-2 cells)

The activation of HSC is central pathophysiological mechanism underlying liver fibrosis. To examine the effects of endogenous expression of Tβ4 in the activated HSCs, we transfected the activated LX-2 cells with a Tβ4-specific siRNA. As shown in Fig 4A, Tβ4 siRNA successfully knocked down Tβ4 mRNA in LX-2 cells (0.074 ± 0.023-fold decrease compared with control siRNA). Also, the level of Tβ4 protein was down-regulated in LX-2 cells treated with Tβ4 siRNA (0.201 ± 0.008-fold decrease compared with control siRNA, **p<0.005). LX-2 cells having the suppressed Tβ4 showed the down-regulated gene expression of myofibroblastic markers, such as transforming growth factor beta (tgf-β, α-sma, collagen type1α1 (col1α1), and vimentin, whereas cells treated with a negative control siRNA had no effect on the activation of HSCs. In addition, typical markers of quiescent HSC, such as Peroxisome proliferator-activated receptor gamma (ppar-γ) and Glial fibrillary acidic protein (gfap), are up-regulated in Tβ4 siRNA-treated cells (Fig 4B). Primary human HSCs transfected with Tβ4 siRNA also showed the decrease of tgf-β, α-sma, and col1α1, and the increase of ppar-γ and gfap, compared to control siRNA or non-treated primary human HSCs (S2 Fig). In the immunofluorescent staining, double positive cells for Tβ4 and α-SMA were rarely detected in the cells treated with Tβ4 siRNA. In addition, Oil Red O staining showed the cytoplasmic lipid droplets, the morphologic hallmark of inactivated HSCs, in the Tβ4-suppressed LX-2 cells (55.4 ± 4.01% of total cells), whereas those lipid droplets were not seen in cells with control siRNA (4.0 ± 1.23% of total cells) (Fig 4C). Therefore, those results demonstrated that the down-regulation of Tβ4 resulted in the inactivation of HSCs.

## Discussion

Chronic liver diseases constitute a global concern. Fibrosis is the essential pathophysiologic feature in chronic liver disease, and the activation of hepatic stellate cell is central to this whole process. Therefore, it is most important to understand its underlying mechanism. Our results demonstrated that the suppression of Tβ4 blocked the activation of HSCs, suggesting that the regulation for Tβ4 expression in HSCs can be an important target for inhibiting hepatic fibrosis.

Recent studies have reported the involvement of Tβ4 in fibrogenesis in several organs. In mice with ureteral obstruction, Tβ4 was shown to have profibrotic effects, whereas N-acetyl-seryl-aspartyl-lysyl-proline (Ac-SDKP), a metabolite of Tβ4, exerted anti-fibrotic actions [10]. Tβ4 was dramatically up-regulated in the obstructed kidney of a tubulointerstitial fibrosis model and glomerulosclerosis. As shown in these studies, Tβ4 is associated with fibrosis, and its effect seems to be dependent on the cellular and patho-physiological conditions, implying that the endogenous expression of Tβ4 may be related to disease progression or prevention. Our study is the first to analyze Tβ4 expression by HSCs from a hepatic subpopulation. Our findings clearly demonstrate that Tβ4 was produced in the activated HSCs, and this endogenous expression of Tβ4 was related to HSC activation leading to liver fibrosis by comprehensive analyses, such as immunostaining as well as QRT-PCR and western blot. Moreover, the increased expression of Tβ4 gene in human livers with advanced (stage 4) fibrosis supported our findings (Fig 1E).

We noticed the different pattern of Tβ4 expression between RNA and protein in CCl_4_-induced liver injury at 6 weeks. Chen et al. [32] and Myöhänen et al. [26] reported that POP activity is up-regulated in normal liver and down-regulated in the damaged liver. In line with their findings, we showed the decreased activity of POP in CCl_4_-treated livers compared to control livers (Fig 2D). These findings suggested that the expression level of Tβ4 in both RNA and protein may remain low in the control mice, because of a higher activity of POP. As the liver is injured, POP activity may be declined, and in turn the amount of Tβ4 protein increases, like the increased Tβ4 protein in CCl_4_-treated liver for 6 weeks. The continuous injury may lead to increasingly the down-regulation of POP activity and the up-regulation of Tβ4, which seems to be enough for the activation of its own signaling pathway, leading to Tβ4 production in both RNA and protein at 10 weeks.

Rojkind et al. [16] showed that the exogenous Tβ4 exerted an anti-fibrotic effect in the acute liver injury model. This result appears to contradict our findings. However, it is possible that exogenous Tβ4 treatment combined with an endogenous increase of Tβ4 may create too high of a Tβ4 concentration, which desensitizes the Tβ4 activation signaling pathway, eventually blocking the transition of HSCs into myofibroblastic HSCs. In addition, we used the chronic liver model by injecting mice with CCl_4_ twice per week for 10 weeks, but Rojkind et al. [16] used the acute injury model made by a single injection of CCl_4_. In addition, Han et al. [33] and Xiao et al. [34] showed the decreased expression of Tβ4 in serum obtained from patients with liver diseases. These findings seem to be different from our data which show the increased expression of Tβ4 in the liver with advanced fibrosis. However, we checked the level of Tβ4 in liver, not in serum. Thus it is possible that the different materials employed in examining the amount of Tβ4 might explain the disparity in Tβ4 expression.

In conclusion, our present study is the first to demonstrate that the activated HSCs express Tβ4, and this expression plays a key role in the regulation of HSC activation during fibrotic injury in the liver. Hence, our data provide insight into the underlying mechanism of Tβ4 in chronic liver disease by identifying the actual role of Tβ4 in liver fibrosis and suggest that Tβ4 may be a new therapeutic target for chronic liver disease treatment.

## Supporting Information

S1 Fig Liver morphology and low magnified images of Tβ4 detection in livers of the CCl_4_-treated mice. H & E (left panel) and Sirius red (middle panel) staining in liver sections from representative control and CCl_4_-treated mice show the distorted liver morphology and collagen deposition, respectively. Immunostaining for Tβ4 (right panel) in liver section from representative CCl_4_ or corn oil-treated mice (X20). (CON: corn oil-treated mice, CCl_4_-treated mice)
(TIF)

S2 Fig Tβ4 siRNA suppresses activation of human primary HSCs. QRT-PCR analysis for tβ4, tgf-β, α-sma, col1α1, ppar-γ and gfap in control siRNA-, Tβ4 siRNA- or no-treated LX-2 cells. Mean±SD results are graphed. Data represent the mean±SD of three independent experiments (*p<0.05; **p<0.005 vs. control siRNA).
(TIF)
