# Supplementary figures and images for: Hepatic Stellate Cells Express Thymosin Beta 4 in Chronically Damaged Liver
Source: PLoS One. 2015 Mar 31;10(3):e0122758. doi: 10.1371/journal.pone.0122758 (PMC4380456; doi:10.1371/journal.pone.0122758)

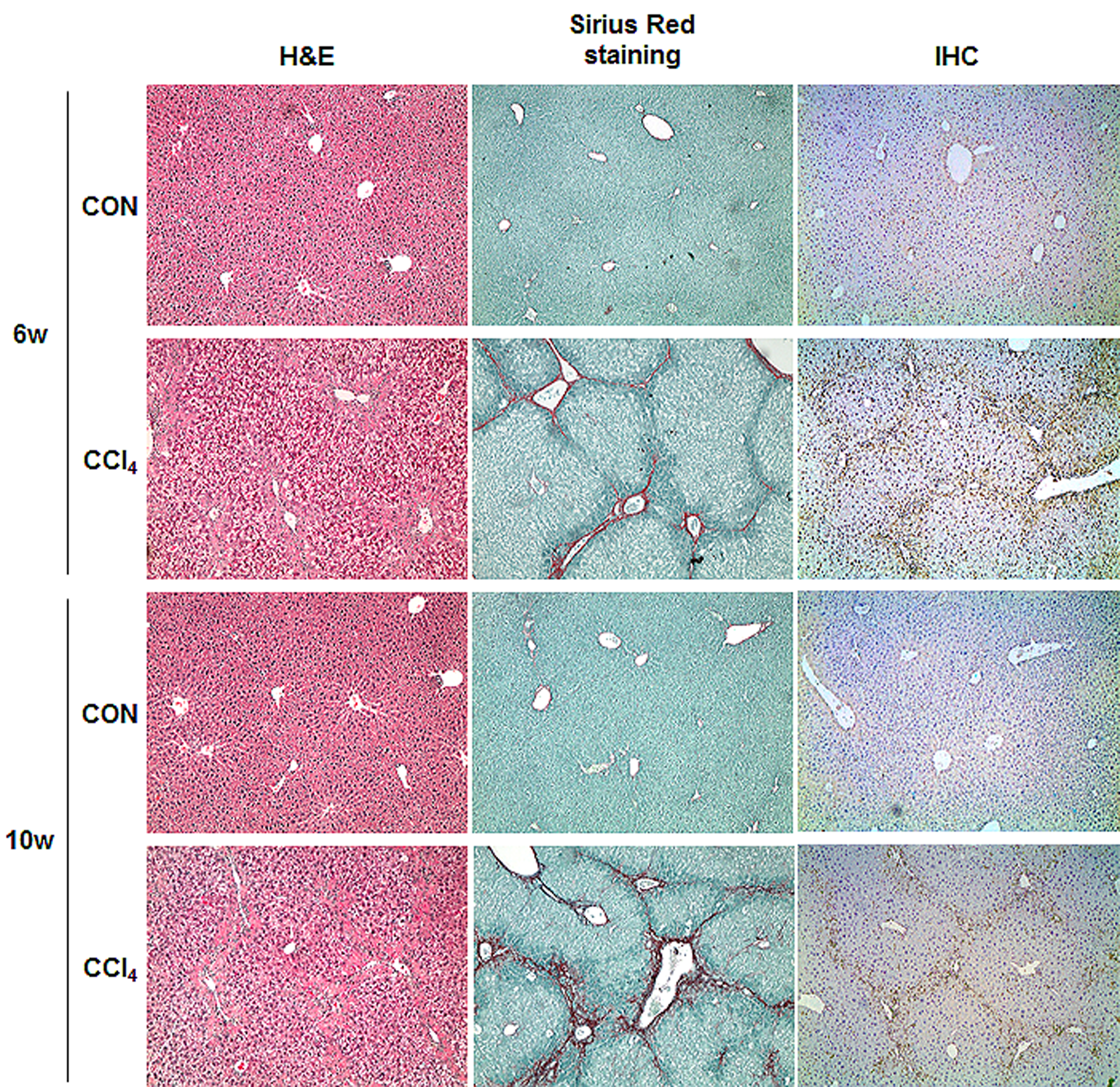

Supplement: S1 Fig — H & E (left panel) and Sirius red (middle panel) staining in liver sections from representative control and CCl4-treated mice show the distorted liver morphology and collagen deposition, respectively. Immunostaining for Tβ4 (right panel) in liver section from representative CCl4 or corn oil-treated mice (X20). (CON: corn oil-treated mice, CCl4-treated mice) (TIF) [file pone.0122758.s001.tif]

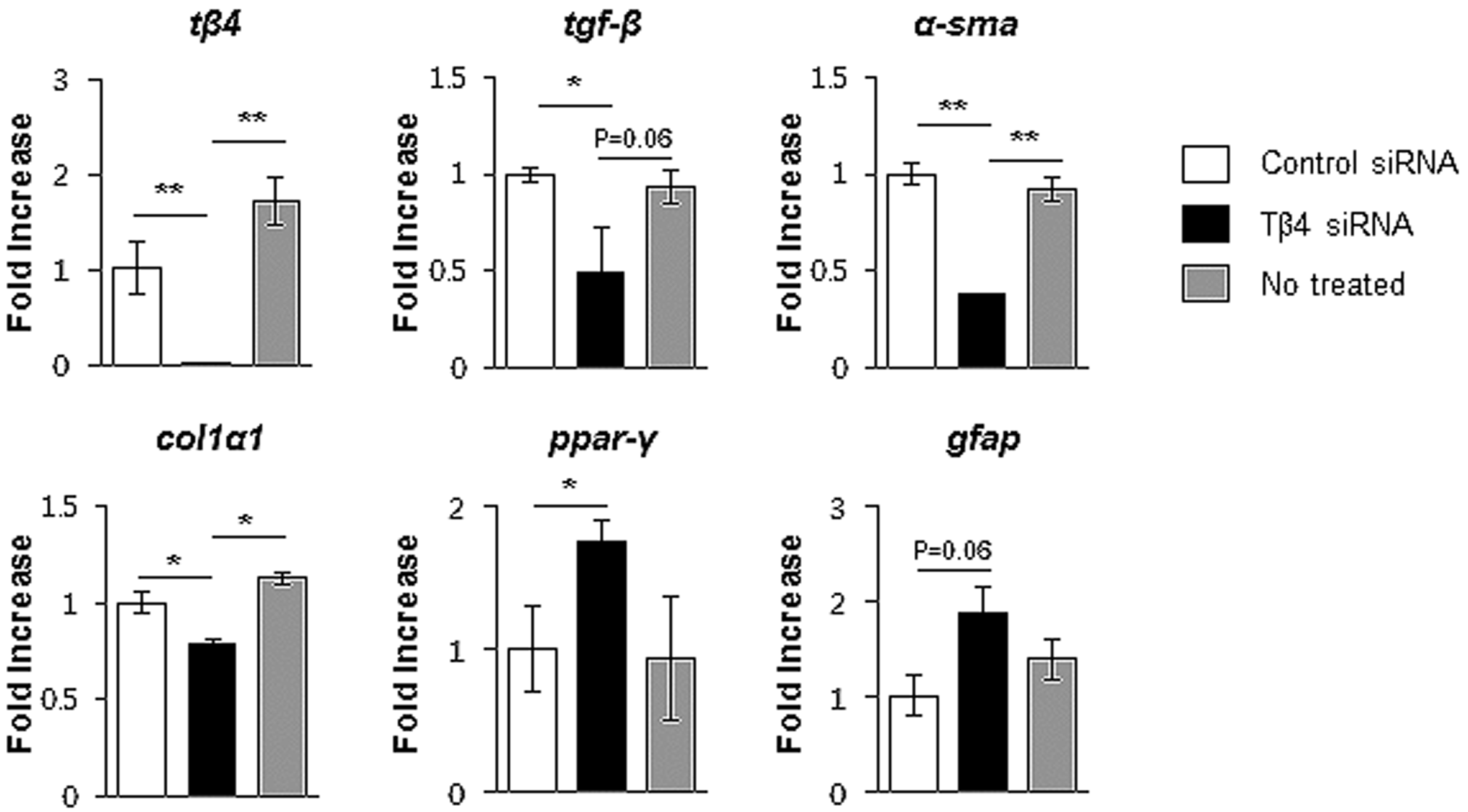

Supplement: S2 Fig — QRT-PCR analysis for tβ4, tgf-β, α-sma, col1α1, ppar-γ and gfap in control siRNA-, Tβ4 siRNA- or no-treated LX-2 cells. Mean±SD results are graphed. Data represent the mean±SD of three independent experiments (*p<0.05; **p<0.005 vs. control siRNA). (TIF) [file pone.0122758.s002.tif]
